# Controllable Synthesis of Tetraethylenepentamine Modified Graphene Foam (TEPA-GF) for the Removal of Lead ions

**DOI:** 10.1038/srep16730

**Published:** 2015-11-19

**Authors:** Zhuo Han, Zhihong Tang, Yuhang Sun, Junhe Yang, Linjie Zhi

**Affiliations:** 1 School of Materials Science and Engineering, University of Shanghai for Science and Technology, Shanghai 200093, China; 2 Department of Mechanical Engineering and Shenzhen Research Institute, Hong Kong Polytechnic University, Hung Hom, Kowloon, Hong Kong, China

## Abstract

3D graphene foam for water purification has become pervasive recently, not only because it has high specific surface area for adsorption capacity, but also it is easily separated from solution after adsorption. However, it is still challenging because it is hard to improve the adsorption capacity as well as maintain the high mechanical strength. To overcome the challenge, Tetraethylenepentamine modified Graphene Foam (TEPA-GF) was synthesized *via* a one-step hydrothermal method by using GO and TEPA as raw materials. TEPA acted as both cross-linker to combine GO sheets together and reductant of GO during hydrothermal process. Results indicated that the resultant hydrogel’s formation was highly dependent on the mass ratio of TEPA to GO, they cross-linked into a stable hydrogel with perfect cylindrical only when M_TEPA_: M_GO_ ≥ 1. What’s more, the highest mechanical strength of GF happened at the mass ratio of M_TEPA_: M_GO_ = 3, which was up to 0.58 kPa. It was worth noting that TEPA-GF demonstrated high adsorption capacity for lead ions, which reached as high as 304.9 mg g^−1^, much higher than that of other absorbents. Furthermore, TEPA-GF was easily separated from water after adsorption of Pb^2+^, making it a great potential material for water purification.

Self-assembly was recognized as one of the most powerful strategies for building nanostructured blocks into three dimensional architectures, it realized the implementation of the superior properties of individual nanomaterial for macroscopic applications^1^,^2^,^3^. Graphene Foam(GF), a typical kind of 3D macroscopic assemblies derived from GO, which consisted of microporous and mesoporous networks and allowed fast access and diffusion of ions and molecules, was potentially used in electrode materials^4^,^5^,^6^,^7^, catalysis^8^, sensers^9^,^10^ and water purification^11^,^12^. Applying 3D graphene either as filter membranes^13^,^14^,^15^,^16^ or blocky adsorbing materials^17^,^18^ in water remediation was more attractive because of high adsorption capacity and easy separation, and adsorption was proved to be more effective because of easy operation and low cost. Therefore, graphene aerogels (GAs) with high mechanical strength, tunable density and volume have been prepared, which showed high adsorption capacity for Pb^2+^ (about 80 mg g^−1^)^19^. In order to improve the absorption capability towards heavy metal ions, GO was hybridized with other materials to form 3D architectures, Zhang *et al.* combined GO with chitosan, the composite aerogel showed an increased sorption capacity towards Pb^2+^ up to 99 mg g^−1 ^20. Surface modifications were also explored for increasing adsorption performance, for example, 3D graphene oxide was fabricated via direct oxidation of CVD graphene foam, which showed a high ability to remove Zn^2+^, Fe^3+^, Pb^2+^ and Cd^2+^ from aqueous solutions, and the adsorption capacity was increased at high PH condition because of the negatively charged functional groups on the foam surface. Unfortunately, the preparation process was complicated and cost was high, which limited the scalable production and further application^21^. Since the negatively charged functional groups were more effective for adsorption of heavy metal ions, amino compound was usually selected to modify porous carbon to improve the adsorption capacity because of the alkaline^22^,^23^,^24^. The same method was also used to modified 3D graphene, a polydopamine-modified graphene hydrogel was obtained by a typical hydrothermal process, which revealed an even higher adsorption capacity towards Pb^2+^ and Cd^2+^, the Pb^2+^ adsorption capacity was up to about 250 mg g^−1^ at the initial concentration of 100 mg ml^−1 ^12. However, less information on the formation process of graphene hydrogel after the addition of dopamine was proposed, and the Pb^2+^ adsorption capacity was expected to improve furtherly.

Herein, 3D TEPA-GF was prepared by a one-step hydrothermal method, the influence of mass ratio of TEPA to GO on the hydrogel’s formation, structure and the adsorption capacity towards Pb^2+^ were investigated. Results demonstrated that the hydrogel formation was highly dependent on the mass ratio of TEPA to GO, and when the concentration of GO was fixed at 2 mg ml^−1^, a stable hydrogel formed only when M_TEPA_: M_GO_ ≥ 1:1. Equally, mechanical strength of TEPA-GF was also affected by the amount of TEPA, the highest mechanical strength was 0.58 kPa when the mass ratio of TEPA to GO was 3:1. Furthermore, TEPA-GF exhibited excellent capability for the adsorption of Pb^2+^, which reached as high as 304.9 mg g^−1^ at the initial Pb^2+^ concentration of 100 mg ml^−1^, much higher than that of pure GF. Therefore, TEPA not only acted as the reductant and modifier of GO during hydrothermal process, but also as active sites for adsorbing Pb^2+^ in the adsorption process.

## Results and Discussion

**Figure 1:**
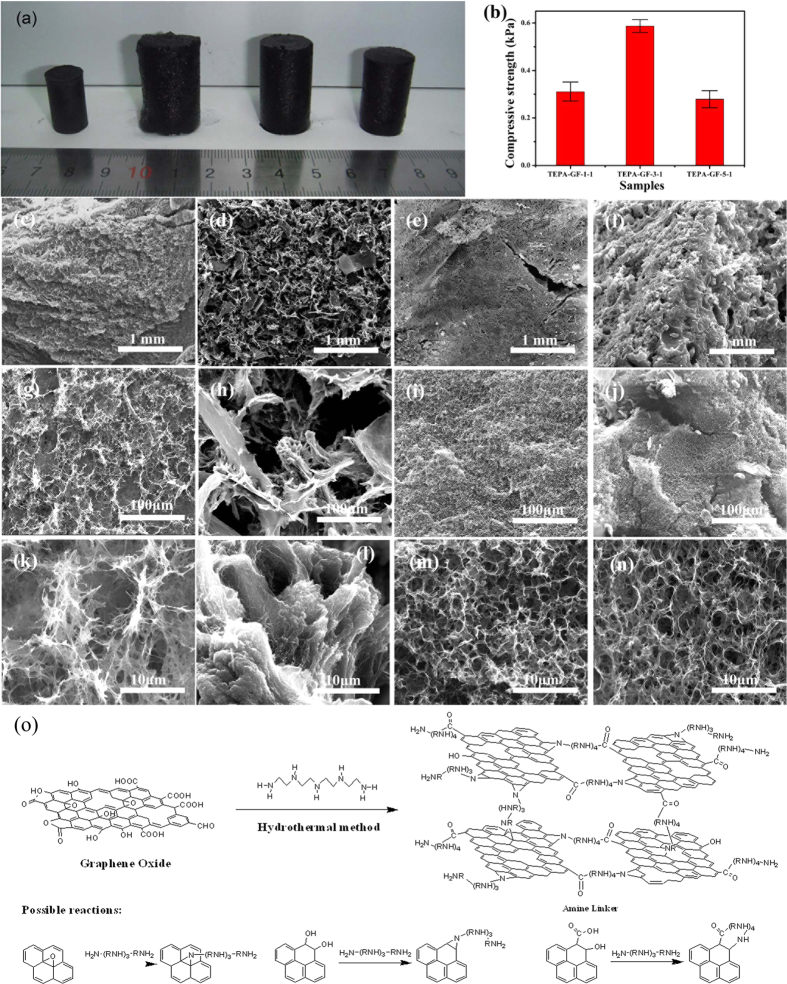
(**a**) Photographs of typical three dimensional GH and TEPA-GH (GH, TEPA-GH-1-1, TEPA-GH-3-1 and TEPA-GH-5-1, from left to right) (**b**) Histograms of the compressive strength of TEPA-GF. (**c**–**n**) SEM images of TEPA-GF. Low and high magnification SEM images and their corresponding models of GF (**c**,**g**,**k**,**o**), TEPA-GF-1-1 (**d**,**h**,**l**,**p**), TEPA-GF-3-1 (**e**,**i**,**m**,**q**) and TEPA-GF-5-1(**f**,**j**,**n**,**r**). (**o**) The proposed mechanism of TEPA-GF’s Assembly during the one step hydrothermal method and possible reaction pathways between GO and TEPA.

Synthesis of 3D graphene hydrogels was initiated under ultrasonic-assisted treatment of the mixture of the GO and TEPA of different amount. When the first drop of TEPA was added into GO solution, a viscous paste was obtained, which was jellified after a hydrothermal process, but TEPA and GO cross-linked into a stable hydrogel unless M_TEPA_: M_GO_ ≥ 1:1. As shown in Fig. 1(a), when pure GO was used, a typical hydrogel with a diameter about 1.0 cm and a height of 1.8 cm was formed. When M_TEPA_: M_GO_ = 1:5, a very soft jelly-like paste with the equivalent shape to Teflon-lined model was formed, but its mechanical strength was so low that could not be taken out completely, when transferred to another container, it cracked to powders (as shown in Figure S1). When M_TEPA_: M_GO_ = 1:1, a stable hydrogel with a diameter of 1.6 cm and height of 2.8 cm formed, which was enlarged greatly compared with GH.

Mechanical strength of TEPA-GF was measured by a *Zwick/Roell* universal testing machine. According to results shown in Fig. 1(b), the compressive strength of the TEPA-GF-1-1 was about 0.31 kPa, slightly smaller than that of GF after ethanol solution treatment (0.41 kPa)^19^, indicating an interaction between TEPA and GO sheets; when the mass ratio reached 3:1, the compressive strength was enhanced to 0.58 kPa; surprisingly, when the ratio arrived at 5:1, the compressive strength was reduced to 0.29 kPa. The results indicated the amount of TEPA played an important role on the formation of TEPA-GF.

More information was observed by scanning electron microscopy (SEM). When GH was directly freeze dried, as shown in Fig. 1(c,g,k), the porous structure formed, and many fractures on the pore walls were observed with magnifications of 1,000 and 10,000 times. Different from which of GF, SEM images of TEPA-GF-1-1 shown in Fig. 1(d,h,l) clearly revealed a partially aggregated micron-sized fractals constructed porous, randomly oriented 3D framework, which led to a weak mechanical strength. When the mass ratio increased to 3:1, as shown in Fig. 1(e,i,m), a continuous macro-porous structure with less fractures formed. Obviously, when the mass ratio reached 5:1, which was shown in Fig. 1(f,j,n), many agglomerations distributed on the surface, and a lot of wide gaps were found with the magnification of 1,000 times, less micron-sized pores were observed and the pore walls became thicker, which led to a weaker mechanical strength. On the basis of these observations, it was predicted that the assembly process of the GO and TEPA via the hydrothermal process, in which GO sheets and proper amount of TEPA interacted together and rendered to a stable 3D architecture.

The possible reaction between TEPA and GO was shown in Fig. 1(o). Under the hydrothermal condition, TEPA would react with carboxylic and hydroxyl functional groups on the edge of GO sheets to form amide-like structure and open epoxides on the plane of GO, and the remaining amino functional group on formed structure further reacted with the oxygen functional groups, then the cross linking between GO sheets happened, at the same time, GO was reduced^25^. Thus, the assembly of GO and TEPA was contributed to van der Waals force, л-л stacking, hydrogen bonding and the covalent bonding. However, when little TEPA was added, although the cross linking happened, only soft hydrogel formed, it was the cross linking only happened for part of GO, and the long distance between functionalized graphene layer and unreacted GO layer weakened van der Waals force, л-л stacking and hydrogen bonding. When M_TEPA_: M_GO_ increased to 1:1, the amount of cross linking increased, and a stable 3D porous structure formed. When M_TEPA_: M_GO_ reached 3:1, almost all of TEPA was reacted with GO, at the main force for forming hydrogel was the covalent bond. While excessive TEPA added, part of cross linking would not happen because TEPA was enough to react with oxygen functional groups on each GO layer, on the other hand, more and more TEPA was adsorbed on the pores of 3D graphene hydrogel by physical adsorption, the severe decrease of nitrogen adsorption amount (Figure S2) and the thicker pore walls of TEPA-GF-5–1 verified the deduce, similarly, the adsorbed TEPA and long distance between functionalized graphene weakened van der Waals force and л-л stacking, TEPA-GF-5–1 with low mechanical strength was obtained.

**Figure 2:**
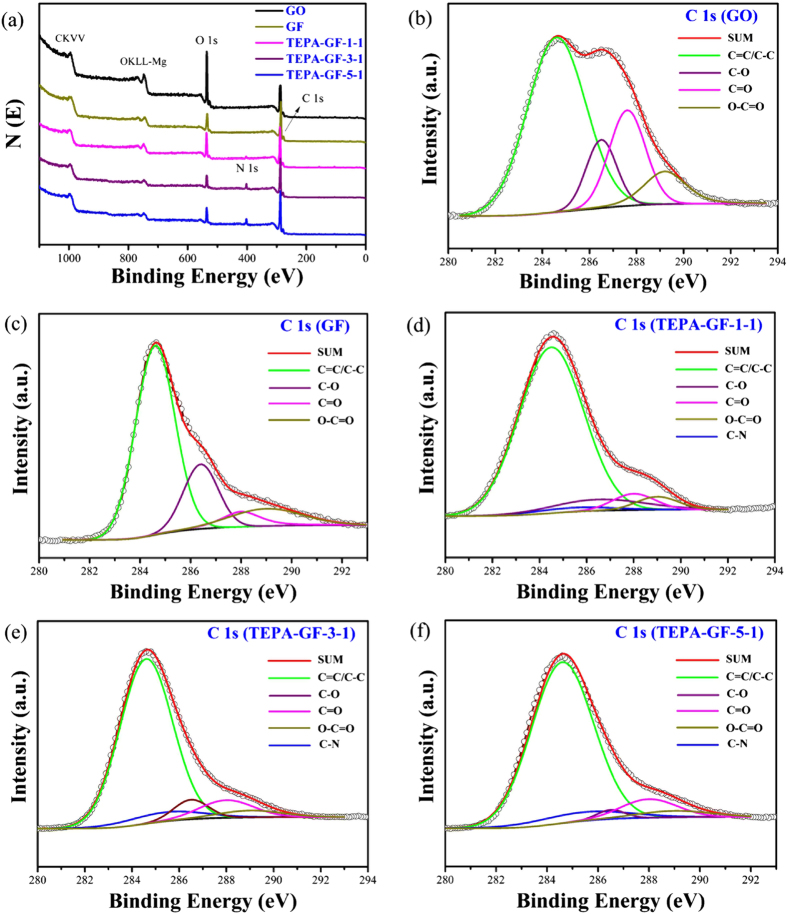
(**a**) XPS survey spectra of GF and TEPA-GF. Deconvoluted XPS C1s spectra of (**b**) GO, (**c**) GF, (**d**) TEPA-GF-1-1, TEPA-GF-3-1 and (**f**) TEPA-GF-5-1.

XPS was used to analyze the elemental compositions and nitrogen bonding in TEPA-GF. Peaks at ~284.6 and 532 eV could be assigned to the binding energies of C1s and O1s, respectively^23^. As shown in Fig. 2, the O1s peak of GF and TEPA-GF became much weaker and the C1s peak became much stronger when compared with that of GO, suggesting the reduction of GO after hydrothermal treatment. In addition, an N1s peak at 399 eV appeared after TEPA participating in the forming of GF, indicating the reaction between TEPA and GO. And the N1s peak increased when increasing the amount of TEPA, as calculated from the spectral, nitrogen content increased from 2.00 to 6.40 at.%, which was contributed to both the reaction of TEPA with GO and the physical adsorption of TEPA on the GF. Meanwhile, the C 1 s XPS spectra of GO, GF was deconvoluted into four distinct peaks at 284.6 eV for C–C/C = C, 286.5 eV for C–O, 287.9 eV for C = O, and 289 eV for O–C = O, respectively^3^,^26^, an additional peak at 285.4 ev^27^ corresponding to the C–N bonds was observed in the C1s spectra of TEPA-GF. The appearance and growing of C-N peak further indicated the successful amino group modification to GF after the one spot hydrothermal process.

**Figure 3:**
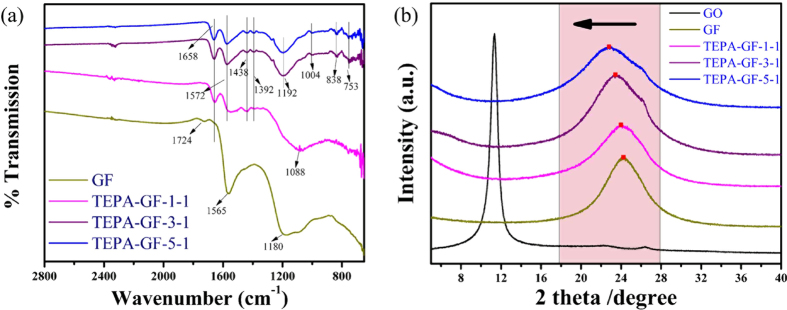
(**a**) FTIR spectral of GF and TEPA-GF, (**b**) XRD spectral of GO, GF and TEPA-GF.

To prove the TEPA has reacted with graphene oxide sheets, FT-IR spectra of GF and TEPA-GF-N-1 were recorded in Fig. 3(a). As shown in the figure, GF had only oxygen functional groups, for example, peaks at 1565 cm^−1^ and 1724 cm^−1^ were assigned to C = O stretching, the peak appeared at 1180 cm^−1^ was due to the bending vibration of the C-O group, another peak at 1091 cm^−1^ was caused by the C–O stretching. As expected, besides the oxygen functional groups, nitrogen functional groups were also appeared in the spectra of the TEPA-GF, peaks appeared at 1438 and 1392 cm^−1^ were caused by symmetric and asymmetric bending vibrations of −NH_2_, another band at 1658 cm^−1^ was attributed to the bending vibration of −N(R)H in TEPA^28^, indicating that TEPA has reacted with the carboxyl or epoxy group on the GO sheets. What’s more, with the increase of TEPA, the peak intensity of these specific functional groups derived from TEPA were enhanced, which suggested that more TEPA had either participated in the reaction or adsorbed on the GF. New peaks appeared at 838 and 753 cm^−1^, due to bonded N-H stretching^29^, also confirmed an interaction between TEPA and the GO sheets.

XRD patterns of GO, GF and TEPA-GF were recorded in Fig. 3(b). The featured diffraction peak of GO appeared at 11.32° (002), calculated by Bragg’s law^30^, corresponding to an interlayer spacing (d-spacing) of 0.781 nm. However, this peak at 2θ = 11.32° entirely disappeared after the hydrothermal reaction due to the deoxidization of oxygen functional groups. Furthermore, a broad diffraction peak centered at around 24.20° (d_002_ of ~ 0.367 nm), 24.00° (d_002_ of ~ 0.370 nm), 23.42° (d_002_ of ~ 0.379 nm) and 22.73° (d_002_ of ~ 0.391 nm) of the graphite (002) plane was observed for GF and TEPA-GF, respectively, indicating stacking of the few-layer reduced graphene oxide sheets during hydrothermal process, what’s more, the enlarged distance between graphene layers indicated the reaction between the amino group and carboxylic and epoxy groups and then the formation of amine-like structures.

**Figure 4:**
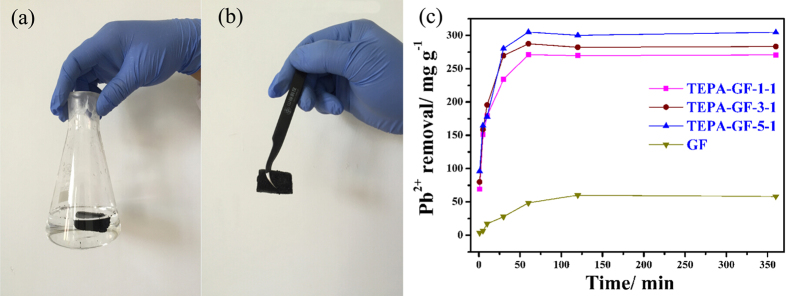
Image of TEPA-GF before (**a**) and after (**b**) adsorption and (**c**) kinetics of Pb^2+^ adsorption of GF and TEPA-GF.

Because of its high specific surface area and abundant functional groups, GO could theoretically adsorb Pb^2+^ as high as 842 mg g^−1^ at 293 K^31^. But it is hard to remove from water due to its excellent hydrophilic property. Alternatively, TEPA-GF could be more suitable for water purifying because of its high specific surface area, porous structure, abundant amine functional groups, high mechanical strength and easy separation. As shown in Fig. 4, TEPA-GF can be easily taken out after adsorption, and TEPA-GF-1-1, TEPA-GF-3-1and TEPA-GF-5-1 exhibited much higher adsorption capability for lead ions than that of GF. Their adsorption capability could be as high as 304.9 mg g^−1^, which is higher than that of GA^19^(our previous work, about 80 mg g^−1^), graphene electrolysis (73.42 mg g^−1^), much better than that of other absorbents^32^, and the comparison of reported adsorption capacity for Pb^2+^ was listed on Table 1. It was worth noting that adsorption amount for Pb^2+^ was increased when the amount of TEPA increased, it was because more TEPA was either reacted or adsorbed on the TEPA-GF, and consequently, more active sites appeared for the adsorption despite the decrease of BET surface area. Meanwhile, the adsorption reached equilibrium in about 50 minutes, which is much faster than GF, and this phenomenon was attributed to its unique macro porous structure and special chemical adsorption. It was worth noting that although there were physically adsorbed TEPA on the TEPA-GF, TEPA was not dissolved in water during adsorption process (Figure S3). The results well demonstrated that the samples were convenient and effective in water purification.

## Conclusion

In summary, Tetraethylenepentamine Modified Graphene Foam (TEPA-GF) was successfully synthesized via a one step hydrothermal method with graphene oxide (GO) and TEPA as raw materials. TEPA not only acted as cross-linker to combine GO sheets together except for van der Waals’ forces, π-π stacking and abundant hydrogen bonds of water during hydrothermal process, but also as reductant of GO. When the mass ratio of TEPA to GO was greater than 1, stable hydrogel was formed, otherwise, only incompact powders were obtained. Continuous porous structures can be obtained for TEPA-GF-3-1, which led to a higher mechanical strength. The resultant samples demonstrated excellent adsorption capability for Pb^2+^, which reached as high as 304.9 mg g^−1^ in 50 minutes, much higher and faster than other carbon absorbent reported.

## Methods

### Preparation of TEPA-GF

GO was chemically exfoliated using a modified Hummer’s method^38^ from pristine graphite flakes, the details of which was described in our previous work^39^. To prepared GO solution with the concentration of 2 mg ml^−1^, 2.2 g graphite oxide was ultra-sonicated in 1 L deionized water for about 2 hours, a brown colloidal suspension was obtained and then which was centrifuged mildly at 3000 rpm for 10 minutes to remove any aggregates or unexfoliated materials.

TEPA-GF was synthesized by a classic hydrothermal method. Typically, 35 ml GO solution of 2 mg ml^−1^ was mixed with TEPA of different amount, then the mixture was put in a Teflon-lined stainless-steel autoclave, stirred, sealed, and hydrothermally treated at 180^ ^°C for 20 hours, after which, the autoclave was naturally cooled to room temperature and the products were washed several times by deionized water, then Tetraethylenepentamine modified Graphene Hydrogels(TEPA-GHs) was obtained, which was labeled as TEPA-GH-N-1, where, N referred to the mass ratio of TEPA to GO. After a directly freeze drying, the corresponding samples were further labelled as TEPA-GF-N-1.

### Characterization

The morphology and structure of obtained samples were observed by scanning electron microscopy (SEM) (FEI Quanta FEG) with an accelerating voltage of 20 kV. Before testing, the specimens were made directly from breaking the cylinder into two parts with fresh fracture surfaces in order to investigate its inner structure. The X ray diffraction (XRD) patterns (5°–80°) were recorded with an XRD operated with Cu Kα radiation (Rigaku Smartlab). Functional groups and chemical bonds were determined by Fourier transform infrared spectroscopy (FTIR) (P.E. Spectrum 100) and X-ray photoelectron spectroscopy (XPS) (PHI 5000 C ESCA). The mechanical strength was measured by a *Zwick/Roell* universal test machine (Materials Testing Machine to 2.5KN, ZWICK). During the measurement, and the compress speed was 1 mm/minute, the process was recorded until the suddenly change. Brunauer−Emmett−Teller (BET) surface areas of the samples were measured by nitrogen adsorption-desorption isotherms performed at 77 K on a Micromeritics ASAP-2020 volumetric adsorption system. The pore size distribution was gained from desorption branch by using Barrett-Joyner-Halenda (BJH) method.

Lead ions’ adsorption experiments were carried out to investigate the adsorption behaviors of GF and TEPA-GF at room temperature. Typically, GF or TEPA-GF (50.0 mg) was put into a flask with 100 mL aqueous solution containing Pb^2+^, and the concentration of Pb^2+^ was 100 mg L^−1^, then the flask was shaken for a period of time or 360 minutes to reach adsorption equilibrium. After adsorption, the GF and TEPA-GF were directly removed from the solution. The concentration of Pb^2+^ after adsorption was determined by an atomic absorption spectrophotometer (AAS, TA5-990). The adsorption experiment of each sample was done for three times, and the concentration of Pb^2+^ after adsorption was averaged. The adsorption capacities of the adsorbents were calculated by the formula.


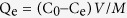


Where C_0_ and C_e_ represent the initial and equilibrium concentrations (mg g^−1^), respectively, where V is the volume of the solutions (mL), and M is the mass of the adsorbents (mg).

## Additional Information

**How to cite this article**: Han, Z. *et al.* Controllable Synthesis of Tetraethylenepentamine Modified Graphene Foam (TEPA-GF) for the Removal of Lead ions. *Sci. Rep.* **5**, 16730; doi: 10.1038/srep16730 (2015).

## Supplementary Material

Supplementary Information

## Figures and Tables

**Table 1:** Adsorption capacity of lead ions with different carbon materials when pH = 7.

Adsorbent	Metalions	Adsorption capacity (mg g^−1^)		Ref.
		C_0_(mg L^−1^)	Qe(mg g^−1^)	
PDA-GH	Pb^2+^	100	250	12
GA	Pb^2+^	100	80	19
CVD graphene via HUMMERS retreatment	Pb^2+^	200	251	32
GO&Fe microwave	Pb^2+^	50	6	33
nitric acid treated MWCNTs	Pb^2+^	60	97.08	34
Single wall CNTs	Pb^2+^	80	32	35
Low-Temperature Exfoliated Graphene	Pb^2+^	80	38.5	36
CNT	Pb^2+^	20	<102.04	37
TEPA-GF	Pb^2+^	100	304.9	This study
