# Supplementary material for: Controllable Synthesis of Tetraethylenepentamine Modified Graphene Foam (TEPA-GF) for the Removal of Lead ions
Source: Sci Rep. 2015 Nov 19;5:16730. doi: 10.1038/srep16730 (PMC4652168; doi:10.1038/srep16730)
Supplement: Supplementary Information [file srep16730-s1.doc]

**Supplementary Information**

**Controllable** **Synthesis of Tetraethylenepentamine Modified Graphene Foam (TEPA-GF) For the Removal of Lead ions**

Zhuo Han^1^, Zhihong Tang^1^*, Yuhang Sun^2^, Junhe Yang^1^*& Linjie Zhi^1^

^1^ School of Materials Science and Engineering, University of Shanghai for Science and Technology, Shanghai, China

^2^ Department of Mechanical Engineering and Shenzhen Research Institute, Hong Kong Polytechnic University, Hung Hom, Kowloon, Hong Kong, China

*Corresponding Author: zhtang@usst.edu.cn；jhyang@usst.edu.cn


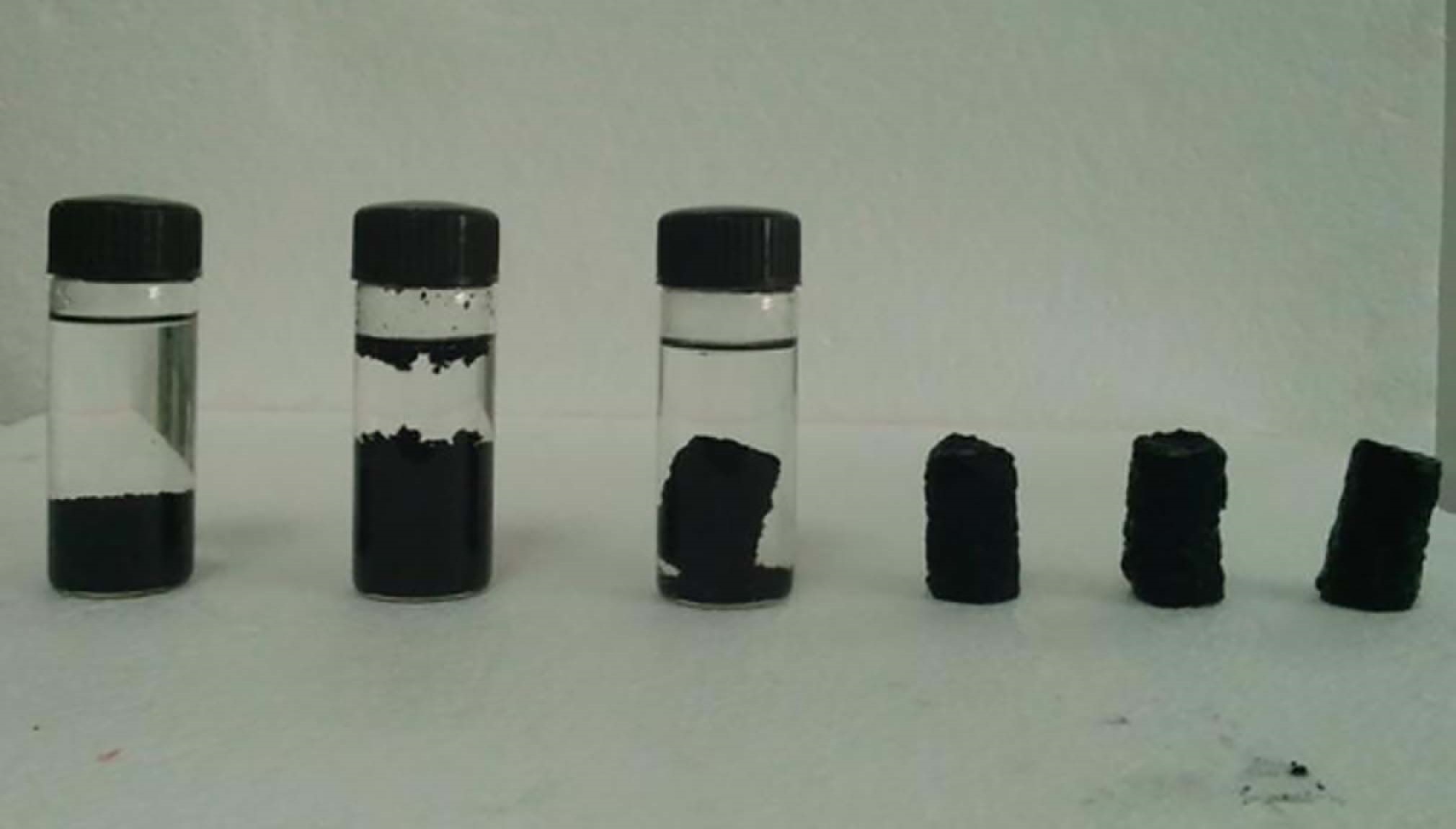


Figure S1 Photographs of a typical three dimensional GH and TEPA-GHs using GO (left) and TEPA of different mass ratio (1:10,1:5,1:1,2:1, 3:1 and 4:1, from 2nd to right) as precursors after hydrothermal process at 180 °C for 20 h.


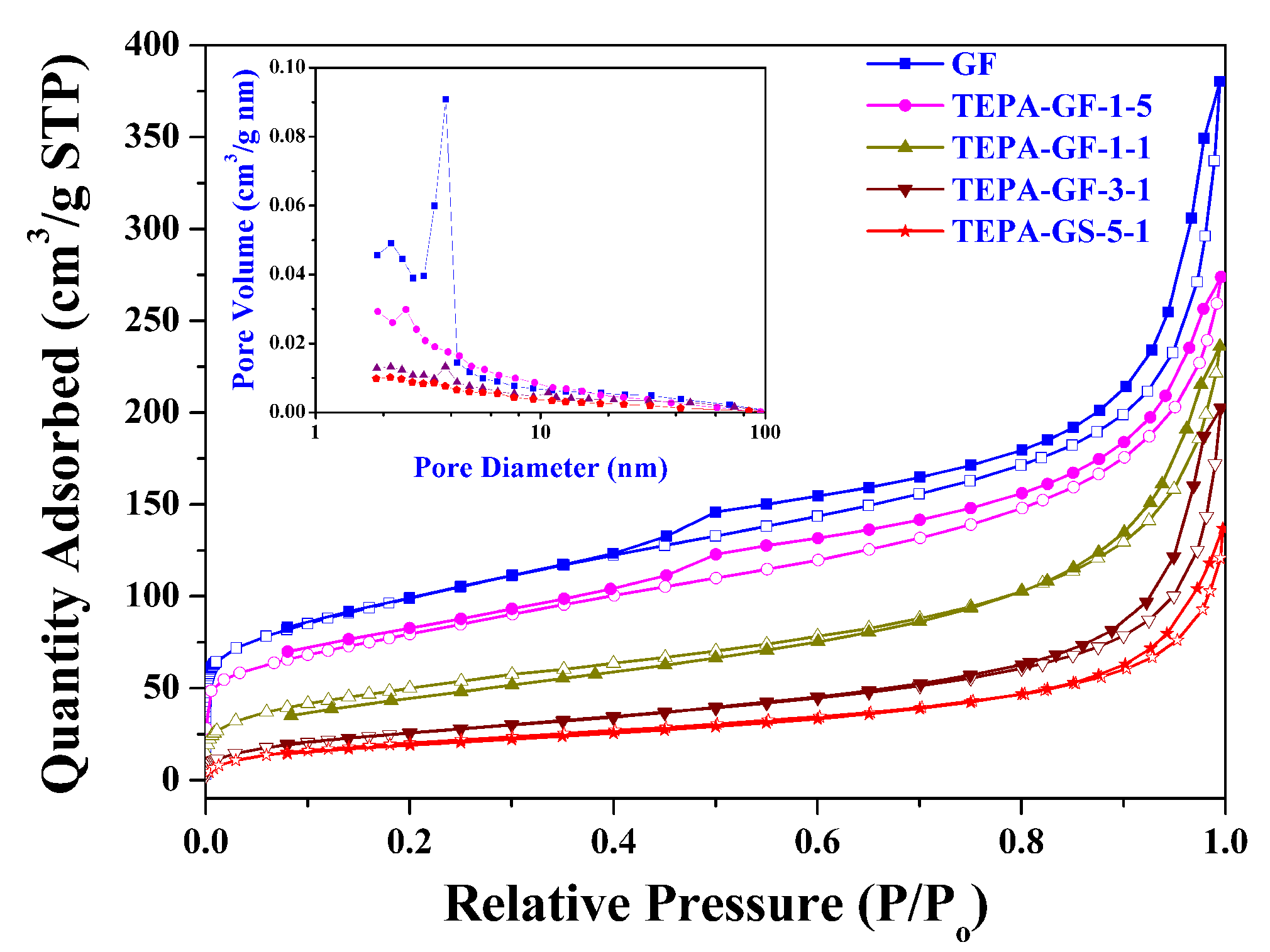
Figure S2 N2 adsorption-desorption isotherms of GF, TEPA-GF-1-5, TEPA-GF-1-1, TEPA-GF-3-1 and TEPA-GF-5-1.


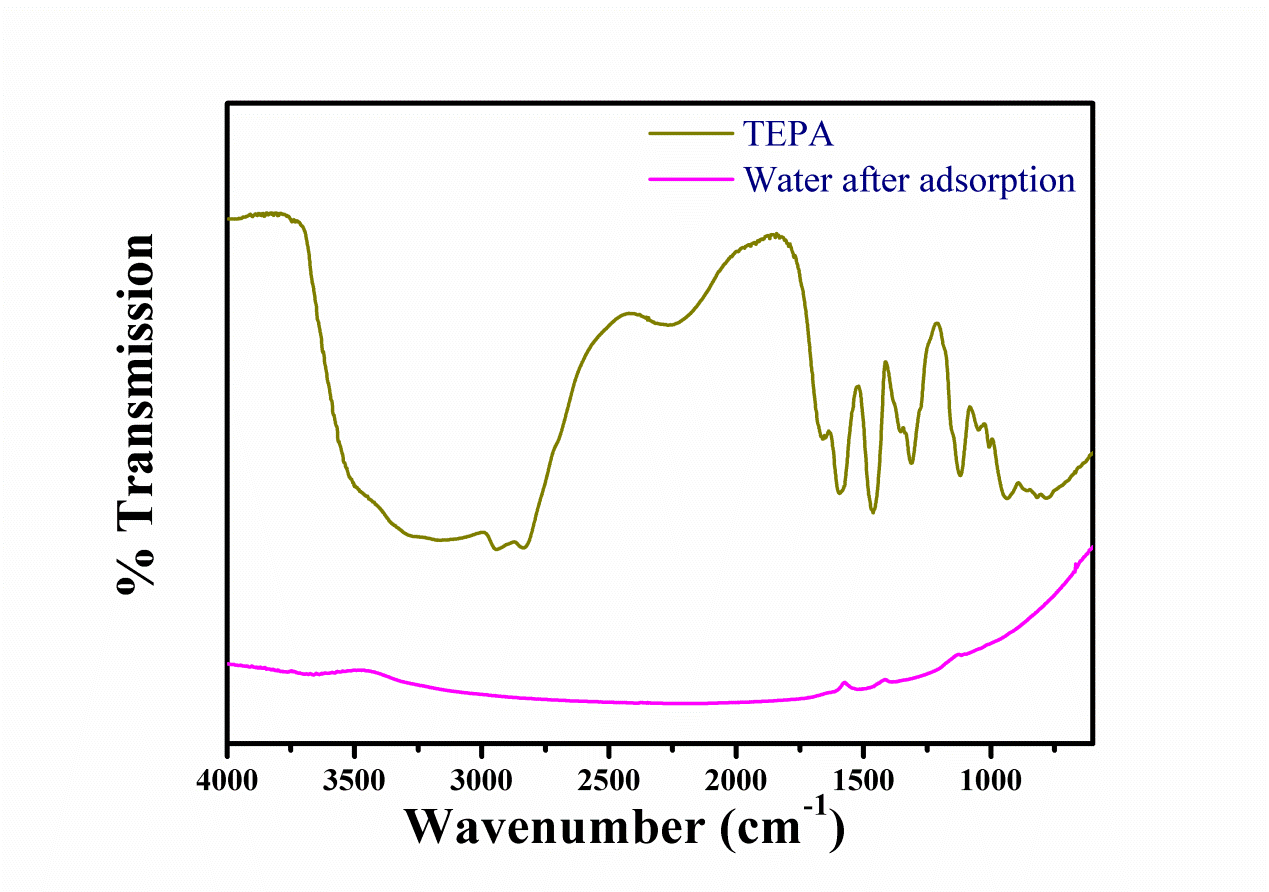


Figure S3 IR spectra of water after Pb^2+^ adsorption of TEPA-GF-5-1, which indicated no residual TEPA existed after test.
